# Formulation and Characterization of a New Injectable Bone Substitute Composed PVA/Borax/CaCO_3_ and Demineralized Bone Matrix

**DOI:** 10.3390/jfb12030046

**Published:** 2021-08-11

**Authors:** Daniela Medrano-David, Aura María Lopera, Martha Elena Londoño, Pedronel Araque-Marín

**Affiliations:** 1 Research Group GIBEC, Life Sciences Faculty, EIA University, Envigado 055420, Colombia; aura.lopera@eia.edu.co (A.M.L.); marta.londono@eia.edu.co (M.E.L.); 2 Research and Innovation Group in Chemical Formulations, Life Sciences Faculty, EIA University, Envigado 055420, Colombia; pedronel.araque@eia.edu.co; 3 CECOLTEC, Medellín 050022, Colombia

**Keywords:** bone tissue regeneration, injectable, bone graft, fracture, osteoblast, bone tissue engineering

## Abstract

The occurrence of bone-related disorders and diseases has dramatically increased in recent years around the world. Demineralized bone matrix (DBM) has been widely used as a bone implant due to its osteoinduction and bioactivity. However, the use of DBM is limited because it is a particulate material, which makes it difficult to manipulate and implant with precision. In addition, these particles are susceptible to migration to other sites. To address this situation, DBM is commonly incorporated into a variety of carriers. An injectable scaffold has advantages over bone grafts or preformed scaffolds, such as the ability to flow and fill a bone defect. The aim of this research was to develop a DBM carrier with such viscoelastic properties in order to obtain an injectable bone substitute (IBS). The developed DBM carrier consisted of a PVA/glycerol network cross-linked with borax and reinforced with CaCO_3_ as a pH neutralizer, porosity generator, and source of Ca. The physicochemical properties were determined by an injectability test, FTIR, SEM, and TGA. Porosity, degradation, bioactivity, possible cytotoxic effect, and proliferation in osteoblasts were also determined. The results showed that the developed material has great potential to be used in bone tissue regeneration.

## 1. Introduction

With the increasing number of bone defects around the world, methods to achieve the rapid and efficient healing of defective bones are attracting attention. In particular, injectable bone substitutes (IBSs) have been identified as suitable biomaterials for bone regeneration [1]. These materials, unlike solid and preformed scaffoldings, can be applied using minimally invasive surgical techniques, thus reducing intervention time, recovery time, and risk of infection [2]. In addition, IBSs facilitate the filling of any irregularly shaped defect, which allows for direct contact between the injected material and the surface of the bone tissue to be treated [3]. Injectable hydrogels have attracted attention, especially because their structures are similar to those of extracellular matrixes [4] and their capacity to promote the adhesion, proliferation, differentiation, and migration of stem cells [5]. In recent years, various injectable hydrogels with good formability and three-dimensional structures have been studied for potential applications in bone tissue engineering [6]. Though these hydrogels have shown great potential for promoting bone regeneration, their poor mechanical properties have limited their further clinical application [7].

**Figure 1:**
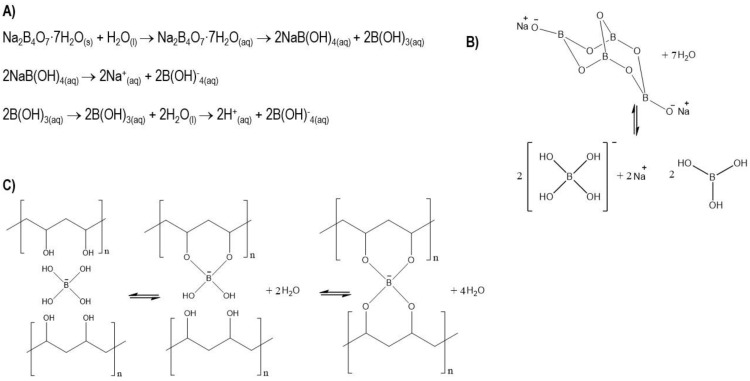
(**A**) Chemical equations of the dissociation of sodium tetraborate (borax) in water; (**B**) model of dissociation; (**C**) physical–chemistry crosslinking process and complexity balance between PVA chains and borate ions.

The choice of the most appropriate polymer depends on biocompatibility, a lack of side effects upon implantation (such as inflammation or allergy), and physical–chemical properties such as degradation and reabsorption [8,9]. Polyvinyl alcohol (PVA) has been extensively studied for its biocompatibility, hydrophilic properties, chemical resistance, and biochemical properties [10,11]. In addition, it is a non-toxic, water-soluble, biocompatible, biodegradable, and safe polymer for medical use [12]. Due to its large number of hydroxyl groups in its chain, combined with excellent water retention, PVA is an ideal choice for self-healing hydrogels, which can self-heal after the material has cracked or broken. This self-repairing design not only extends the life of the material but also restores and/or maintains its original performance [13]. Despite the possible toxicity that borax can generate [14], it is a good candidate for physically crosslinking PVA chains through hydrogen bonds and reversible diol-borate esters [15]. It should be noted that the toxicities reported from borax correspond to concentrations much higher than those used in this work. The specific mechanism of the complexity of PVA with borax is as follows: a borax mol is dissociated into 2 mol of borate ions and borate acid, as shown in Figure 1. The complexity mechanism of PVA–borate ions has two balances governed by interactions with hydrogen, monodiol, and didiol complexities [16]. The complex of PVA and borax in aqueous solutions has been thoroughly studied for several decades [17,18]. Its compatibility with biological environments has been proven, and its properties have allowed hydrogels to be considered promising for various applications such as tissue engineering [19], drug release [20], and wound dressing [21]. The PVA–borax hydrogel has been received the attention in this research because it can form a self-healing hydrogel that could increase the stability of an injectable system during and after injection.

Recently, through the introduction of bioactive inorganic compounds, many composite hydrogel scaffolds with enhanced mechanical properties have been developed for bone regeneration [22]. DBM is currently considered a good candidate in clinical practice due to its osteoinductive and osteoconductive properties [23,24,25,26]. For this reason, this research combined the physicochemical properties of PVA-borax hydrogel with the osteoconductive and osteoinductive properties of DBM to create an injectable bone substitute. In addition, CaCO_3_ was incorporated to increase osteoconductivity and neutralize pH [27,28,29]. The injectability of the developed materials was evaluated, and they were also characterized by FTIR, SEM, and TGA. The porosity was determined by the liquid displacement technique. The in vitro degradation kinetics was determined by the immersion technique in simulated body fluid (SBF), and this fluid was also used for the evaluation of bioactivity by analyzing the surface of the materials after immersion in SBF and then looking for calcium phosphates. Finally, the cytotoxicity of the IBS was evaluated by an MTT assay, and cell proliferation was evaluated with an Alamar Blue kit.

## 2. Materials and Methods

### 2.1. Materials

DBM with particle sizes between 125 and 300 μm was obtained from the Tissue Bank Foundation, Medellín, Colombia. PVA, -CH_2_CHOH- (130,000 Mw and 99% hydrolyzed), calcium carbonate (CaCO_3_), and borax (Na_2_B_4_O_7_·7H_2_O) were acquired from Sigma Aldrich Co (St. Louis, MO, USA). Glycerol was acquired in JM chemicals (Medellín, Antioquia, Colombia). For SBF, we used sodium chloride (NaCl), sodium bicarbonate (NaHCO_3_), potassium chloride (KCl), dipotassium hydrogen phosphate trihydrate (K_2_HPO_4_·3H_2_O), magnesium chloride hexahydrate (MgCl_2_·6H_2_O), hydrochloric acid (HCl), calcium chloride (CaCl_2_), sodium sulfate (Na_2_SO_4_), and basic TRIS purchased from Sigma Aldrich Co (St. Louis, MO, USA). All solutions were prepared with Milli-Q Water with an electrical conductivity of approximately 0.18 μS.

Human osteosarcoma osteoblasts (Saos-2 ATCC^®^ HTB-85™ cell line, (Manassas, VA, USA), McCoy’s 5A culture medium with bicarbonate, L-glutamine, fetal bovine serum (FBS), and antibiotics (penicillin–streptomycin) were used for biological evaluation. Phosphate-buffered saline (PBS) and MTT (thiazolyl blue tetrazolium bromide) from Thermo Fisher (Waltham, MA, USA), were used for cytotoxicity evaluation. Alamar Blue Invitrogen from Thermo Fisher (Waltham, MA, USA), was used for the evaluation of cell proliferation.

### 2.2. Methods

#### 2.2.1. Injectable Bone Substitute Preparation

A liquid phase composed of a PVA/glycerol solution in a 1/3 ratio and 3% borax was mechanically mixed with a solid phase of DBM and calcium carbonate. Final injectable bone substitute samples were added in syringes for ease of application. Table 1 specifies the composition percentages of each sample or formulation.

#### 2.2.2. Injectability

To measure the extrusion force necessary to inject the substitutes, an equal volume (1 mL) of each of the formulations was filled into a 2 mL syringe without a needle that was fixed vertically and perpendicularly to the clamp in an Instron mechanical test machine (model 3345), at 26 °C with a load cell of 5000 N and a speed of 5 mm/min. Each experiment was repeated three times. This methodology was based on the methodology employed by Dyah Hikmawati and co-authors [30].

(1) $$\mathrm{Injectability}\;\left(\%\right)=\left(\frac{\mathrm{mass}\;\mathrm{extruded}\;\mathrm{from}\;\mathrm{the}\;\mathrm{syringe}}{\mathrm{total}\;\mathrm{mass}\;\mathrm{before}\;\mathrm{injection}}\right)\times 100\%$$

#### 2.2.3. Physicochemical Characterization

Scanning electron microscopy (Phenom ProX Desktop SEM, Eindhoven, The Netherlands) was used to examine the morphology and microstructure of the sample, and then the pore sizes were measured with Image J. Using the liquid displacement methodology (hexane) implemented by Rutusmita Mishra et al., the percentage of porosity was measured [31]. Fourier transform infrared ray (FTIR–ATR, Perkin Elmer Spectrum 100, Shelton, CT, USA) spectroscopy was performed in the range of 4000–550 cm^−1^ with a resolution of 4 cm^−1^ to evaluate the binding of all the components of the injectable system. To reveal the thermal behavior of the injectable formulations, a thermogravimetric analyzer (TA Instruments, model TGA Q500, New Castle, UK) was used. The heating ramp was 5 °C/min from 30 to 1200 °C under a nitrogen atmosphere.

#### 2.2.4. Degradation

To evaluate the degradation of the injectable bone substitute, samples of the different formulations were immersed in plastic containers containing 5 mL of SBF at a temperature of 37 °C for periods of 3, 7, 14, 21, and 28 days. The samples were measured for pH (acidity test) and weighed before immersion and on each proposed day of observation (according to ASTM F2900-11, which describes mass loss as a degradation kinetics test for biomedical hydrogels). The test was carried out with SBF prepared in the Biomaterials laboratory according to the Kokubo protocol [32]. At the end of each proposed period, the samples were frozen and subsequently lyophilized to guarantee that the moisture acquired during the immersion time was eliminated [33]. Weight loss (Wl) was calculated as shown in Equation (2), where W0 denotes the initial dry weight of the samples and Wd represents the weight of the dry samples after the programmed immersion time [34].

(2) $$\mathrm{Weight}\;\mathrm{loss}\;\left(\%\right)=\frac{W0-\mathrm{Wd}}{\mathrm{Wd}}\;\times \;100$$

#### 2.2.5. Bioactivity Evaluation

To determine the bioactivity property of the IBS, we used the methodology proposed by Kokubo, which requires SBF with pH adjusted to 7.4 to immerse the samples in for a specified time at a temperature of 37 °C. The samples after completing 3, 7, and 14 days of immersion in SBF were left to dry in a vacuum desiccator at 37 °C, and, finally, they were analyzed by means of SEM to analyze the surface of the material and identify the possible formation of apatite crystals [32,35]. The composition of the material formed on the surface was evaluated by means of energy dispersive X-ray spectroscopy (Phenom ProSuite v2.8.0 EDX, Eindhoven, The Netherlands).

#### 2.2.6. Cell Culture

The Saos-2 cell line (ATCC^®^ HTB-85™, Manassas, VA, USA) human osteosarcoma osteoblasts were grown in McCoy’s 5A culture medium reconstituted with 2.2 g/L of bicarbonate, 10 mL/L of L-glutamine, and 10% supplemented with FBS and 1% antibiotics (penicillin–streptomycin). The culture medium was changed every two or three days, and the incubation conditions were 37 °C and 5% CO_2_.

#### 2.2.7. MTT Assay

The methodology used to analyze the cytotoxicity of particulate F1, F2, and DBM was an indirect test performed according to ISO 10993-5 with SaOS-2 cells similar to human osteoblasts (although SaOS-2 cells are an osteosarcoma cell line, their suitability as an osteoblast cell model has been widely demonstrated) [36]. Each of the three samples was immersed in McCoy’s 5A culture medium for 24 h and then centrifuged, and the supernatant was used to treat the osteoblast monolayer at 90% confluence with a cell concentration of 20,000 cells/well in a 96-well plate for 72 h. Then, the medium was replaced with the MTT stock solution (12 mM) previously diluted to 10% *v/v* in a fresh medium, as the manufacturer recommends. After being incubated for 4 h at 37 °C, cell culture was protected from light, the medium with MTT was removed and the formazan crystals were dissolved by adding 200 µL of DMSO to each well. The MTT reduction was quantified by measuring the light absorbance with an ELISA microplate reader at 570 nm for 10 s with orbital motion.

#### 2.2.8. Cell Proliferation

The osteoblast monolayer was used for the evaluation of cell proliferation using the Alamar Blue kit from Thermo Fisher, which evaluates mitochondrial ability to reduce resazurin in the fluorescent product resorufin. First, 50,000 cells/well were seeded in a 96-well dish. Before carrying out the assay, an Alamar Blue medium was prepared by mixing the medium with an Alamar Blue solution in a ratio of 10:1. After treatment with the formulations for 24, 48, and 72 h, the medium was discarded and replaced by the medium with Alamar Blue. The microplates were incubated at 37 °C for 3 h, and the fluorescence was measured using a BIO-RAD X ELISA reader (Thermo Fisher, Waltham, MA, USA) at an excitation wavelength of 535 nm and an emission wavelength of 590 nm. Cell viability was calculated using the ratio between the fluorescence of the treated cells and the fluorescence of the control cells.

#### 2.2.9. Statistical Analysis

After testing the normal distribution of the data with the Shapiro–Wilk test, the groups were compared using ANOVA, and then the post hoc test (Tukey) was used. Significant differences were verified by Student’s *t*-test. Values of *p* < 0.05 were considered statistically significant. All measurements in the different tests were collected at least in triplicate and expressed as mean ± standard deviation (SD). Statistical analysis was performed with Minitab 19 software (State College, PA, USA).

## 3. Results

### 3.1. Injectability

Injectable hydrogels are a class of hydrogels that can be extruded through a syringe [37]. The objective of the test was to determine the force necessary to extrude the material through a syringe and to calculated the percentage of that injectability using Equation (1). The results are summarized in Table 2.

**Figure 2:**
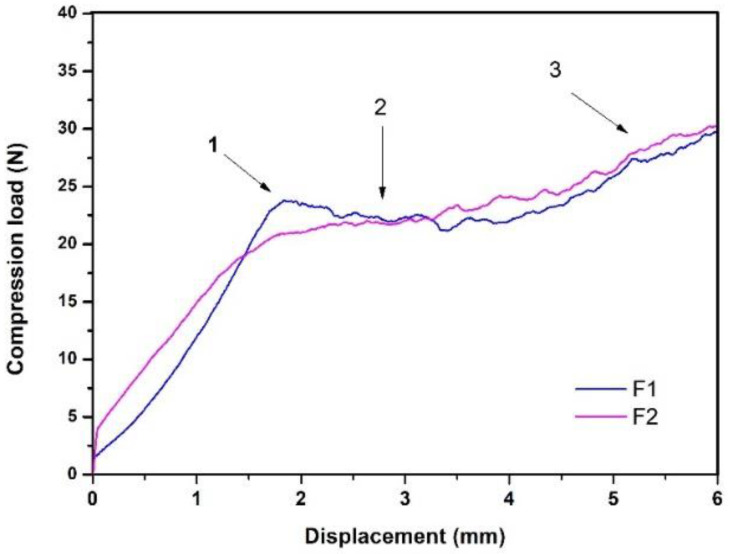
Injectability behavior of F1 and F2.

Because Formulation 3 presented a low percentage of injectability and a very high injection force, it was excluded from this research. The injection behavior of formulations 1 (F1) and 2 (F2) is shown in Figure 2, where the *y*-axis represents the compression stress (N) exerted by the load cell on the syringe plunger and the *x*-axis the displacement made by this same. This figure identifies three relevant events in injection behavior, which have been reported by other authors [38,39], and are indicated from left to right as follows:

1. “Overexertion” or “overshoot”: initial overstrain is required to overcome hydraulic pressure inside the syringe.
2. Platea or plateau: this area indicates a greater presence of solids; in this case, the plateau in this case.
3. The maximum effort at the end of the injection: this indicates the point of mechanical resistance exerted by the plunger against the end of the syringe.

### 3.2. Physicochemical Characterization

**Figure 3:**
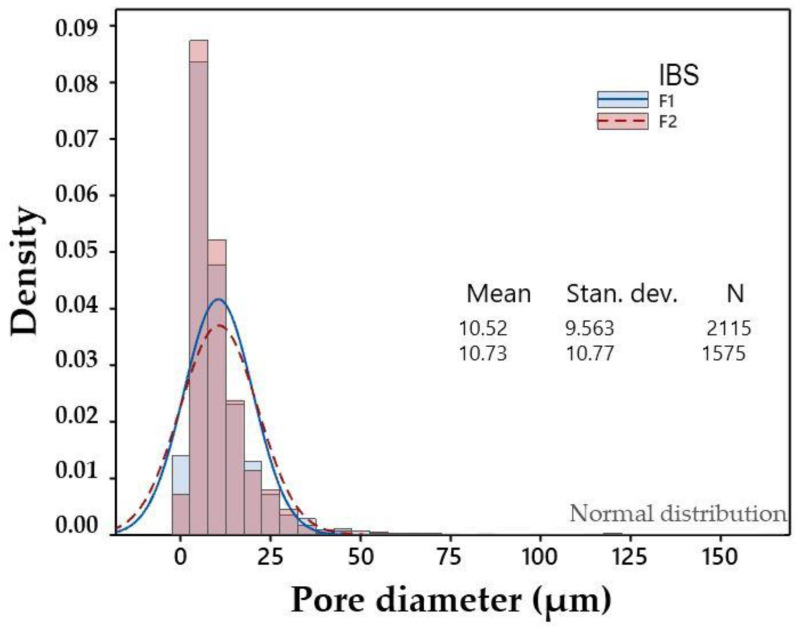
Histogram of pore diameters.

One of the reasons for incorporating calcium carbonate is because this material produces a powerful and prolonged neutralization of acidity, forming CaCl_2_ and CO_2_ [40]. In this work, it was also decided to take advantage of the release of CO_2_ generated by effervescence [41] that happens when the system was prepared to generate porosity in the material. The results of the liquid displacement test showed that F2 had a higher percentage of porosity compared to F1, with values of 53.19 ± 0.01 and 50.8 ± 0.1, respectively. It is important to note that F2 and F1 were composed of 5% and 2% CaCO_3_, respectively, so it is possible to suggest that as the content of CaCO_3_ in the system increased, the porosity also increased. The pore sizes obtained by Image J of both injectable formulations were analyzed by ANOVA. It was found that there were no statistically significant differences between F1 and F2, which suggests that when decreasing the percentage of CaCO_3_ from 5% to 2%, the pore size did not significantly vary; however, the percentage of porosity did. A histogram of pore size values is shown in Figure 3.

**Figure 4:**
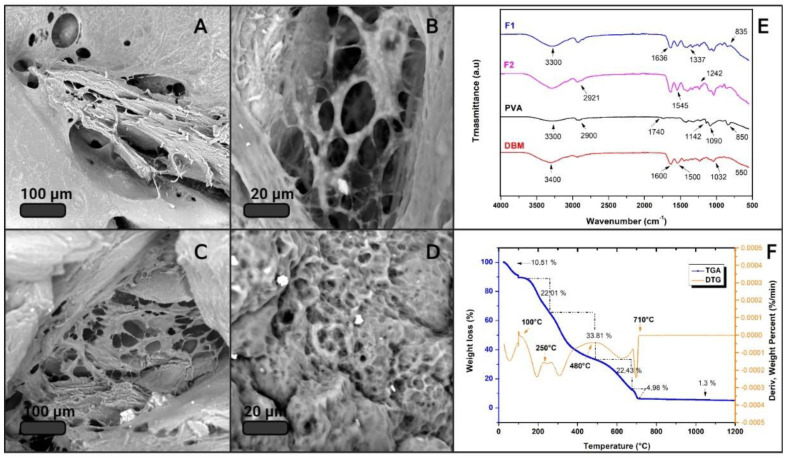
Physicochemical characterization. (**A**,**B**) SEM of F1; (**C**,**D**) SEM of F2; (**E**) FTIR spectrum of the main components, F1 and F2; (**F**) TGA.

Figure 4 shows the results of the physicochemical characterization of F1 and F2. SEM micrographs show, in all cases, a heterogeneous surface with pores and clusters of cubic morphology, which correspond to calcium carbonate. This was verified by EDS. CaCO_3_ clusters could provide roughness to the surface of the material, which could favor cell adhesion [42]; however, this hypothesis must be verified by cell adhesion assays for these composite materials.

An FTIR analysis was performed on the main IBS components to characterize the most representative functional groups of each material. Figure 4E shows the IR spectra of PVA, DBM, F1, and F2. The red spectrum corresponds to the DBM, showing a band around 3400 cm^−1^ associated with OH groups. The most representative DBM peaks were identified at 1600 and 1500 cm^−1^, which are attributed to vibrations of the amide groups, of collagen and other proteins, respectively [43]. The band at 1032 cm^−1^ and the beginning of the band at 550 cm^−1^ are associated with the bending mode caused by vibration of phosphate group PO_4_^3−^, confirming the presence of calcium phosphates, a mineral component of bone that remains as a residual mineral component after the demineralization of DBM [44]. The black spectrum corresponding to PVA shows the characteristic bands of polyvinyl alcohol. There appears to be a band around 2900 cm^−1^ attributed to the stretching of the C–H alkyl, and the broadband around 3300 cm^−1^ corresponds to the hydroxyl group. A band was also identified around 1090 cm^−1^, and a small one at 1740 cm^−1^ [45], according to the literature [46,47,48], can mainly be attributed to the crystallinity of PVA and related to the carboxyl stretch band (C=O). The band at 1142 cm^−1^ has been used as an evaluation tool for the structure of the semi-crystalline PVA [49]. The spectra of F1 and F2 appear to be an overlap with the spectra of PVA and DBM. In the two spectra, one can observe broadband at a height of 3300 cm^−1^ that is attributed to moisture absorption and a small one at 1637 cm^−1^ that is attributed to hydroxyl vibrations. The peaks at 2921, 1436, 1242, 1035, and 8345 cm^−1^ can be assigned to the vibrations of CH_2_, double bond C=C, and C–O [50]. The band at 1042 cm^−1^ is related to asymmetric P–O stress due to PO_4_^3−^. The vibrations around 1180 cm^−1^ mainly correspond to minerals; in this case, the source of calcium was CaCO_3_ [51]. The band that appears at 1337 cm^−1^ is due to the O–H bending of the hydroxyl groups that interact with borate ions or form intermolecular hydrogen bonds [52], thus confirming the crosslinking between PVA by means of borax [53].

It is important to know a material’s thermal stability to determine under what conditions it can be handled, stored, and sterilized. Therefore, the samples that met the criteria for injectability underwent TGA. As expected, the samples did not vary much from each other, since their composition was similar. Figure 4F shows the result of the thermogravimetric evaluation from 25 to 1200 °C corresponding to F1, where the blue curve represents the TGA and the orange one represents the DTG. The TGA result for F2 is attached in the Supplementary Materials. The thermogram clearly identifies that the weight decreased as the temperature increased, and six thermal events stand out. The first event around 100 °C with a weight loss of 5.5% can be attributed to the loss of moisture and volatile functional groups. This same event was reported by J. Barrera and co-authors, and their thermal analysis of PVA reveals a percentage of surface water of between 3 and 5% [54]. The PVA degradation process occurs in three steps [55]. The first occurs due to the degradation of the side chain of the polymer [56]; in this case, it was identified to be around 225 °C with a weight loss of 21.81%. Then, at approximately 390 °C, the degradation of 26% of the material was identified and associated with the dehydration of the hydroxyl groups of the main chain of PVA [57]. Around 480 °C, a weight loss of 5.5% occurred, which can be attributed to the third stage of decomposition of PVA, due to the C–C main polymer chain broken; this is called carbonation [58]. The localized processes after the one mentioned above correspond to the degradation of proteins and fats of high molecular weight present in the DBM, as well as to the loss of carbon dioxide, which was generated from the thermal decomposition of CaCO_3_ into CaO and CO_2_ [59]. Calcium oxide was the residual material in the sample, as it did not degrade at 1200 °C. Finally, the percentage of total weight loss was 84.5%, and the residual percentage corresponded to possible phosphates present in the DBM and calcium oxides of CaCO_3_ [60].

### 3.3. Degradation

**Figure 5:**
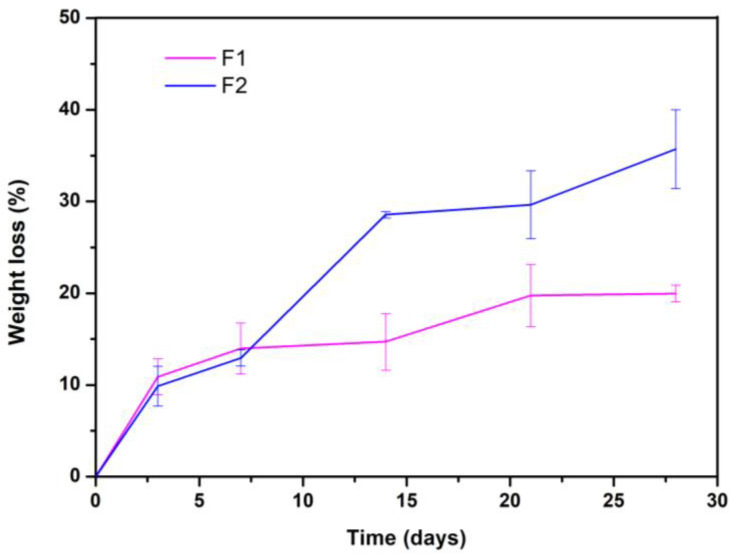
Degradation kinetics of the two evaluated formulations.

The rate of degradation is one of the most important indicators to evaluate the quality of scaffolds since too fast or too slow degradation seriously affects the application of the scaffold [61]. Figure 5 shows the weight loss of F1 and F2 concerning the immersion time in days.

F2 lost about 35% of its weight after 28 days of immersion, and the rapid degradation of the material is an unfavorable aspect concerning the application that this research sought [61,62]. However, F1 lost only 20% of its weight after 28 days of immersion, suggesting that it is more stable than F2. Finally, when all the samples were weighed, i.e., on days 3, 7, 14, 21, and 28, the pH was also measured. The results showed that until the end of the experiment, there were no significant differences in the values, given that all the formulations (at all times) maintained the pH of the fluid at 7.4 ± 0.2.

### 3.4. Bioactivity Evaluation

**Figure 6:**
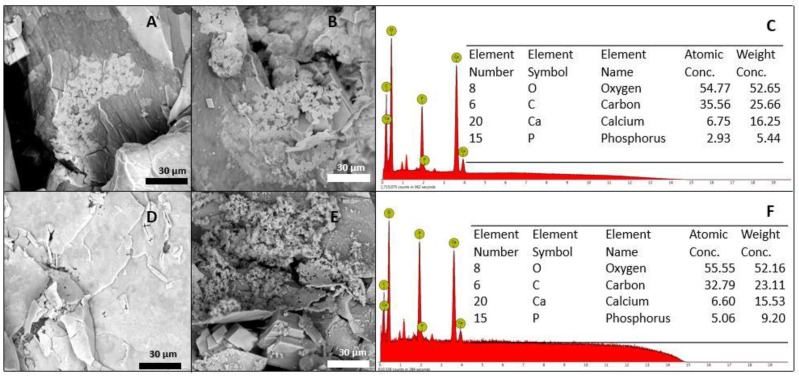
Bioactivity evaluation after 7 days of immersion: (**A**) F1, (**B**) F2, and (**C**) EDS. Bioactivity evaluation after 14 days of immersion: (**D**) F1, (**E**) F2, and (**F**) EDS.

The suitability of the present composite was examined by immersion in SBF for two weeks, followed by an analysis of the resulting surface layer by SEM–EDS. In Figure 6, the SEM micrographs of the two formulations that meet the injectability criteria, before their immersion in SBF, are shown. After 7 days of immersion in SBF, the surface of these samples showed significant changes. In Figure 6D,E, the beginning of the formation of calcium phosphate clusters can be observed, and the Ca/P ratio was determined by EDS and found in an approximate range between 2.3 and 2.9. The calcium/phosphorus ratio of apatite is reportedly between 1.5 and 2.6 [63], but the evaluated formulations showed a layer formation of calcium phosphates with a slightly higher Ca/P ratio. This increase in the Ca/P ratio of the samples, compared to those reported in the literature, could be explained by the presence of calcium ions contributed by CaCO_3_. After 14 days, the apatite clusters were able to more uniformly deposit, forming a predominant layer on the surface of the material and reaching an approximate Ca/P ratio of 1.30, which is comparable to the Ca/P ratio of the amorphous calcium phosphates present in natural bone [64]. The formulations (at all times) maintained the pH of the fluid at 7.4 ± 0.2.

### 3.5. MTT Assay

**Figure 7:**
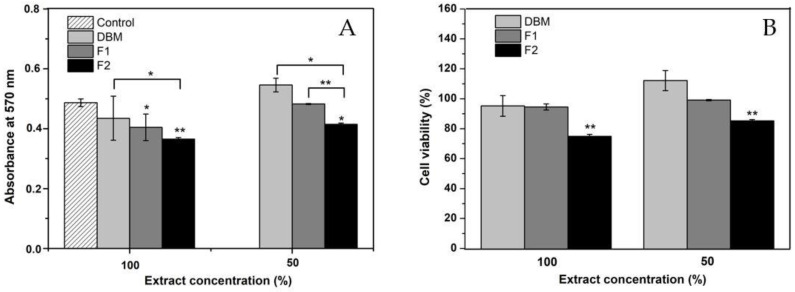
(**A**) Results of the MTT evaluation in absorbance at 570 nm; (**B**) MTT results in the percentage of cell viability. * *p* < 0.05, ** and, *p* < 0.01 compared to the control. Error bars represent ± standard deviation.

The possible cytotoxic effect of DBM and the two injectable formulations developed in this work was evaluated using the MTT test, a colorimetric method that quantifies the activity of succinic dehydrogenase (SDH) [65] and is widely used as an indication of cellular mitochondrial function [66]. The results are shown in Figure 7B. For each treatment, the extract was tested at 100% and its dilution at 50% in a fresh medium. The absorbance values at 570 nm were analyzed by ANOVA and a post hoc test (Tukey’s test). The statistical analysis of all treatments and the control resulted in a *p*-value of >0.05 by ANOVA, which denotes the rejection of the null hypothesis that presumes equality of variances (see Figure 7A). The Tukey grouping with a confidence level of 95%, in the results of the extracts at 100%, showed that F2 was significantly different from the control and the other treatments. However, the cell viability was higher than 70% in all cases, so it can be concluded that none of the materials generated a cytotoxic effect on the osteoblasts, even with the 100% extract.

### 3.6. Cell Proliferation

**Figure 8:**
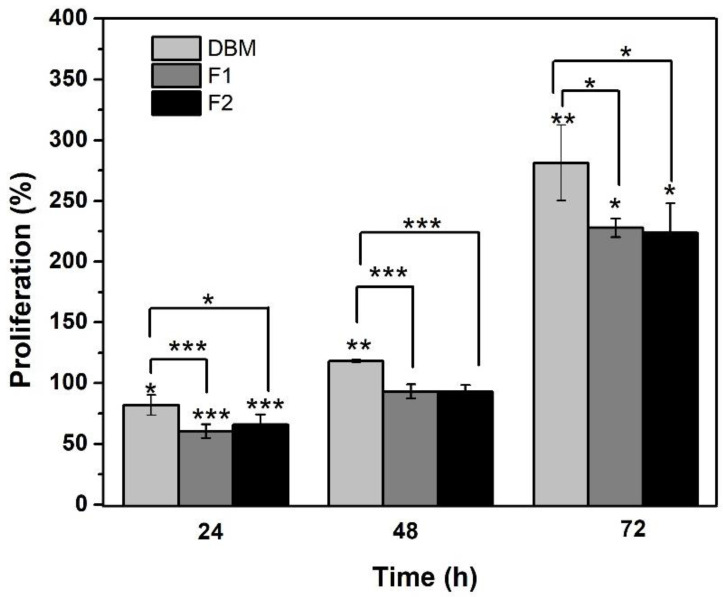
Results of Alamar Blue in percentage of cell proliferation. * *p* < 0.05, ** *p* < 0.01, and *** *p* ≤ 0.001 compared to the control. Error bars represent ± standard deviation.

The proliferation of Saos-2 osteoblasts treated with DBM and the injectable formulations was evaluated by quantifying the metabolic activity using the Alamar Blue kit at 24, 48, and 72 h of incubation. The results are shown in Figure 8, where the *Y*-axis represents the percentage of cell proliferation and the *X*-axis represents that of incubation in hours. At 24 h, a lag phase in the culture was identified, since all the treatments significantly reduced cell viability. This event was attributed to cell adaptation [67] because proliferation was later evidenced. These results support those obtained by the MTT assay, where none of the formulations were shown to have a cytotoxic effect on cells.

## 4. Discussion

Neves and co-authors indicated that for a bone substitute to be considered injectable, the force to extrude it through a syringe must be less than 100 N [68]. Under this criterion, it is possible to conclude that except for Formulation 3, all the evaluated formulations are injectable and possess an injectability of greater than 90%. The percentages of injectability of these two formulations, when compared with those reported by Dorati and his collaborators, are high. They reported an injectable material with an injectability between 70 and 75% and considered it acceptable for this type of application [69].

The formulations referred to in this work as F1 and F2 showed ease to be extruded by a syringe, which is a great advantage in minimally invasive surgeries that reduce the risks of infections in the patient, decrease the time of the intervention, and facilitate implantation to the surgeon [70]. IBSs can be used to treat bone defects and/or irregularly shaped fractures because they can take bone defect’s exact shape. This means that they can have closer or direct contact with the entire surface of the tissue to be treated, as well as avoid the complications of block or preform grafts that frequently present necrosis and mucosal perforation [71]. In addition, when the material is injected, it is not wasted because it directly fills the bone defect. Formulation 3 presented an injectability of 59% ± 19, showing its low homogeneity at the time of injection; the force necessary to overcome the hydraulic pressure inside the syringe exceeded 100 N, and this varied with the displacement. This could be explained by a phenomenon reported in the literature known as phase separation in injectable materials [72]. This phenomenon occurs more frequently in injectables that have an aqueous phase, where a part of this can lodge near the needle with some precipitated solids and generate a greater initial resistance to injection, thus making the material in the body of the syringe less homogeneous [72]. Therefore, the authors of this research only continued evaluating F1 and F2.

A scaffold cannot provide appropriate microenvironments to protect cell proliferation and differentiation if the rate of degradation is too rapid. A slower rate of degradation can cause the scaffold residue to become foreign tissue or even induce an inflammatory response that will make it difficult to repair a bone defect site [62]. With this, it could be concluded that F1, in comparison to F2, would be the most appropriate to be used in bone regeneration due to its structural stability and controlled degradation rate. Bao et al. manufactured a Pluronic diacrylate hydrogel F127 and incorporated nano-CaCO_3_ to improve bone regeneration; the results of their degradation test showed that the weight loss of the hydrogel decreased by approximately 15%. By incorporating calcium carbonate, the hydrogel maintained approximately 85% of its mass after 40 days of immersion in PBS, which, according to the authors, is beneficial in supporting bone tissue regeneration [7]. The pH remained balanced during the immersion time due to the presence of CaCO_3_, which has been widely reported and implemented to neutralize pH in degradation processes [73]. Furthermore, calcium carbonate improves cellular reactions relative to pure polymers, since it is a source of calcium ions that promote bone regeneration [74].

Bioactivity is the ability of a material to chemically interact with the living tissues of the body; this ability results from the release of ions until the formation of a mineralized layer on the surface of the material [75]. Bioactive materials must be able to form hydroxyapatite (HA) on their surface during immersion in SBF [76]. HA formation on an implant surface is considered a key factor for the creation of bone junction and the index of bone-forming capacity [77]. The formulations evaluated in this investigation were able to form calcium phosphates on their surfaces after immersion in SBF. These suggest by carrying DBM in this PVA-borax system, its bioactive property is not affected. Furthermore, the IBS’s proposed were shown to be bioactive. The bioactivity of these IBS is made possible by the free calcium ions, which bind with the phosphate ions in the SBF solution and then precipitate again at the active sites on the surface of the material. This demonstrates the capacity for biomineralization, which is an essential factor in promoting osteogenic bone-binding capacity for bone repair materials [35]. The calcium phosphates present in materials can cause bone induction through their high capacity to bind proteins (including growth factors), their specific architectures, or the calcification of living surfaces. For this reason, calcium phosphates (Ca_3_(PO_4_)_2_) are widely used in bone repair and regeneration due to their osteoconductive and bioactive nature (osteointegration) [78].

In the case of the DBM, this proliferative effect was expected in human osteoblasts since DBM is composed of collagen and bone morphogenetic proteins. Though the collagen matrix provides an osteoconductive effect, the BMPs provide osteoinductivity [79,80]. Our results are consistent with those reported by Adkisson and his co-authors, who reported that human Saos-2 osteosarcoma cells proliferate in response to DBM [81]. Furthermore, they quantitatively correlated this proliferative activity with the osteoinductive capacity in vivo, but this correlation was not significant [82,83]. Jordan M. Katz and his collaborators also made efforts to determine the correlation of specific growth factors and cell proliferation in vitro versus ectopic bone formation in vivo. They found a significant positive correlation, but it was not sufficient enough to extrapolate the in vitro results with those obtained in vivo [84]. It should be noted that osteoinduction refers to the ability of a material to stimulate the differentiation of a cell towards a lineage of osteoblasts that will deposit minerals [85]. The high expression of alkaline phosphatase (ALP) is an early indicator of the differentiation and maturation of osteoblasts [86], as well as one of the most reported evaluation metrics of in vitro osteoinduction [87]. Based on the abovementioned ideas, we can conclude that when DBM is carried in the carrier polymeric system developed in this work, its proliferative capacity in human osteoblasts is not affected. It could also be suggested that the injectable material developed in this research has great potential to be an osteoinductive bone substitute, but to conclude this, quantitative bone differentiation tests such as ALP and semi-quantitative tests such as red staining should be performed [88,89].

## 5. Conclusions

In this research, a polymeric system was developed to carry DBM and be used as an injectable bone substitute that promises regenerative characteristics of bone tissue. The injectability test showed that two formulations capable of being extruded by a needleless syringe were developed, with a compression force of less than 100 N. It was also found that the injectability of both formulations was greater than 90%. The developed material is microporous, with a porosity of around 50% that could favor protein adsorption and nutrient transport processes. When immersing the samples in SBF and determining the degradation of the formulations, it was established that F1 was the formulation with the highest cohesion, that is, it was the one that showed less particle loss and greater stability in contact with the fluid. This formulation degraded by approximately 20%. after spending 28 days submerged in SBF. It was also found that none of the formulations altered the pH of the SBF during the experiment. From the evaluation of the bioactivity, it can be determined that the developed IBS comprises a bioactive material since all of the evaluated formulations promote the formation of calcium phosphates on its surface. This means that the bioactive capacity of the DBM is not affected when it is integrated into the carrier system. By quantifying the mitochondrial activity of osteoblasts treated with DBM and injectable formulations using the MTT assay, it was concluded that none of these materials induce cytotoxicity in cells. The results of the quantification of the metabolic activity of osteoblasts using the Alamar Blue kit showed that when DBM is carried in the developed carrier system, its proliferative property in osteoblasts is not affected. Furthermore, this proliferative effect of injectable formulations suggests that these materials are osteoinductive. However, more evidence is needed before stating conclusions, e.g., by quantifying markers of differentiation in osteoblasts and biomineralization.

## Figures and Tables

**Table 1:** The composition of formulations evaluated in this study.

Approx. Weight Composition (%)						
Formulation	Powders			Liquids		
	DBM	CaCO_3_	PVA	Glycerol	Borax	Water
1	26	2	2	6.5	0.5	63
2	25	5	2	6.5	0.5	61
3	24	5	3.6	6.5	0.4	60.5

**Table 2:** Injectability test results.

Formulation	Max. Compression Load (N)	Injectability (%)
1	50 ± 2	93 ± 1
2	13 ± 1	94 ± 1
3	193 ± 29	59 ± 19

## Data Availability

Data is contained within the article or Supplementary Material.

## Supplementary Materials

The following are available online at https://www.mdpi.com/article/10.3390/jfb12030046/s1.

## References

[1] González Ocampo J.I., Machado de Paula M.M., Bassous N.J., Lobo A.O., Ossa Orozco C.P., Webster T.J. (2019). Osteoblast responses to injectable bone substitutes of kappa-carrageenan and nano hydroxyapatite. Acta Biomater..

[2] Li Y., Rodrigues J., Tomás H. (2012). Injectable and biodegradable hydrogels: Gelation, biodegradation and biomedical applications. Chem. Soc. Rev..

[3] Kretlow J.D., Klouda L., Mikos A.G. (2007). Injectable matrices and scaffolds for drug delivery in tissue engineering. Adv. Drug Deliv. Rev..

[4] Wang L., Lu R., Hou J., Nan X., Xia Y., Guo Y., Meng K., Xu C., Wang X., Zhao B. (2020). Application of injectable silk fibroin/graphene oxide hydrogel combined with bone marrow mesenchymal stem cells in bone tissue engineering. Colloids Surf. A: Physicochem. Eng. Asp..

[5] Ren K., Cui H., Xu Q., He C., Li G., Chen X. (2016). Injectable Polypeptide Hydrogels with Tunable Microenvironment for 3D Spreading and Chondrogenic Differentiation of Bone-Marrow-Derived Mesenchymal Stem Cells. Biomacromolecules.

[6] Re F., Sartore L., Moulisova V., Cantini M., Almici C., Bianchetti A., Chinello C., Dey K., Agnelli S., Manferdini C. (2019). 3D gelatin-chitosan hybrid hydrogels combined with human platelet lysate highly support human mesenchymal stem cell proliferation and osteogenic differentiation. J. Tissue Eng..

[7] Bao Z., Gu Z., Xu J., Zhao M., Liu G., Wu J. (2020). Acid-responsive composite hydrogel platform with space-controllable stiffness and calcium supply for enhanced bone regeneration. Chem. Eng. J..

[8] Vukajlovic D., Parker J., Bretcanu O., Novakovic K. (2018). Chitosan based polymer/bioglass composites for tissue engineering applications. Mater. Sci. Eng. C.

[9] Sundaram M.N., Amirthalingam S., Mony U., Varma P.K., Jayakumar R. (2019). Injectable chitosan-nano bioglass composite hemostatic hydrogel for effective bleeding control. Int. J. Biol. Macromol..

[10] Mohebali A., Abdouss M., Afshar Taromi F. (2020). Fabrication of biocompatible antibacterial nanowafers based on HNT/PVA nanocomposites loaded with minocycline for burn wound dressing. Mater. Sci. Eng. C.

[11] Batool S., Hussain Z., Niazi M.B.K., Liaqat U., Afzal M. (2019). Biogenic synthesis of silver nanoparticles and evaluation of physical and antimicrobial properties of Ag/PVA/starch nanocomposites hydrogel membranes for wound dressing application. J. Drug Deliv. Sci. Technol..

[12] Das P., Ojah N., Kandimalla R., Mohan K., Gogoi D., Dolui S.K., Choudhury A.J. (2018). Surface modification of electrospun PVA/chitosan nanofibers by dielectric barrier discharge plasma at atmospheric pressure and studies of their mechanical properties and biocompatibility. Int. J. Biol. Macromol..

[13] Ai J., Li K., Li J., Yu F., Ma J. (2021). Super flexible, fatigue resistant, self-healing PVA/xylan/borax hydrogel with dual-crosslinked network. Int. J. Biol. Macromol..

[14] Hadrup N., Frederiksen M., Sharma A.K. (2021). Toxicity of boric acid, borax and other boron containing compounds: A review. Regul. Toxicol. Pharmacol..

[15] Koga K., Takada A., Nemoto N. (1999). Dynamic light scattering and dynamic viscoelasticity of poly(vinyl alcohol) in aqueous borax solutions. 5. Temperature effects. Macromolecules.

[16] Mahjoub H.F., Zammali M., Abbes C., Othman T. (2019). Microrheological study of PVA/borax physical gels: Effect of chain length and elastic reinforcement by sodium hydroxide addition. J. Mol. Liq..

[17] Li J., Liu Y., Chen Q. (2020). Conformation of dilute poly(vinyl alcohol)-borax complex by asymmetric flow field-flow fractionation. J. Chromatogr. A.

[18] Lin H.-L., Liu Y.-F., Yu T.L., Liu W.-H., Rwei S.-P. (2005). Light scattering and viscoelasticity study of poly(vinyl alcohol)-borax aqueous solutions and gels. Polymer.

[19] Zhao C., Qazvini N.T., Sadati M., Zeng Z., Huang S., De La Lastra A.L., Zhang L., Feng Y., Liu W., Huang B. (2019). A pH-Triggered, Self-Assembled, and Bioprintable Hybrid Hydrogel Scaffold for Mesenchymal Stem Cell Based Bone Tissue Engineering. ACS Appl. Mater. Interfaces.

[20] Liang Y., Zhao X., Ma P.X., Guo B., Du Y., Han X. (2019). pH-responsive injectable hydrogels with mucosal adhesiveness based on chitosan-grafted-dihydrocaffeic acid and oxidized pullulan for localized drug delivery. J. Colloid Interface Sci..

[21] Kondiah P.J., Choonara Y.E., Kondiah P.P.D., Marimuthu T., Kumar P., du Toit L.C., Pillay V. (2016). A Review of Injectable Polymeric Hydrogel Systems for Application in Bone Tissue Engineering. Molecules.

[22] Hoffman A.S. (2012). Hydrogels for biomedical applications. Adv. Drug Deliv. Rev..

[23] Kobayashi H., Kato M., Taguchi T., Ikoma T., Miyashita H., Shimmura S., Tsubota K., Tanaka J. (2004). Collagen immobilized PVA hydrogel-hydroxyapatite composites prepared by kneading methods as a material for peripheral cuff of artificial cornea. Mater. Sci. Eng. C.

[24] Hameed N., Glattauer V., Ramshaw J.A.M. (2015). Evaluation of polyvinyl alcohol composite membranes containing collagen and bone particles. J. Mech. Behav. Biomed. Mater..

[25] Kinard L.A., Dahlin R.L., Lam J., Lu S., Lee E.J., Kasper F.K., Mikos A.G. (2014). Synthetic biodegradable hydrogel delivery of demineralized bone matrix for bone augmentation in a rat model. Acta Biomater..

[26] Tian M., Yang Z., Kuwahara K., Nimni M.E., Wan C., Han B. (2012). Delivery of demineralized bone matrix powder using a thermogelling chitosan carrier. Acta Biomater..

[27] Dizaj S.M., Barzegar-Jalali M., Zarrintan M.H., Adibkia K., Lotfipour F. (2015). Calcium Carbonate Nanoparticles; Potential in Bone and Tooth Disorders. Pharm. Sci..

[28] He F., Zhang J., Yang F., Zhu J., Tian X., Chen X. (2015). In vitro degradation and cell response of calcium carbonate composite ceramic in comparison with other synthetic bone substitute materials. Mater. Sci. Eng. C.

[29] Eglin D., Mortisen D., Alini M. (2009). Degradation of synthetic polymeric scaffolds for bone and cartilage tissue repairs. Soft Matter.

[30] Hikmawati D., Maulida H.N., Putra A.P., Budiatin A.S., Syahrom A. (2019). Synthesis and Characterization of Nanohydroxyapatite-Gelatin Composite with Streptomycin as Antituberculosis Injectable Bone Substitute. Int. J. Biomater..

[31] Mishra R., Varshney R., Das N., Sircar D., Roy P. (2019). Synthesis and characterization of gelatin-PVP polymer composite scaffold for potential application in bone tissue engineering. Eur. Polym. J..

[32] Kokubo T., Takadama H. (2006). How useful is SBF in predicting in vivo bone bioactivity?. Biomaterials.

[33] Yang Z., Zhao F., Zhang W., Yang Z., Luo M., Liu L., Cao X., Chen D., Chen X. (2021). Degradable photothermal bioactive glass composite hydrogel for the sequential treatment of tumor-related bone defects: From anti-tumor to repairing bone defects. Chem. Eng. J..

[34] Jing X., Mi H.Y., Salick M.R., Cordie T., Crone W.C., Peng X.F., Turng L.S. (2014). Morphology, mechanical properties, and shape memory effects of poly(lactic acid)/thermoplastic polyurethane blend scaffolds prepared by thermally induced phase separation. J. Cell. Plast..

[35] Zhang X., Chen Y., Han J., Mo J., Dong P., Zhuo Y., Feng Y. (2019). Biocompatiable silk fibroin/carboxymethyl chitosan/strontium substituted hydroxyapatite/cellulose nanocrystal composite scaffolds for bone tissue engineering. Int. J. Biol. Macromol..

[36] Prideaux M., Wijenayaka A.R., Kumarasinghe D.D., Ormsby R.T., Evdokiou A., Findlay D.M., Atkins G.J. (2014). SaOS2 osteosarcoma cells as an in vitro model for studying the transition of human osteoblasts to osteocytes. Calcif. Tissue Int..

[37] Pal A., Vernon B.L., Nikkhah M. (2018). Therapeutic neovascularization promoted by injectable hydrogels. Bioact. Mater..

[38] Zhang J., Liu W., Gauthier O., Sourice S., Pilet P., Rethore G., Khairoun K., Bouler J.M., Tancret F., Weiss P. (2016). A simple and effective approach to prepare injectable macroporous calcium phosphate cement for bone repair: Syringe-foaming using a viscous hydrophilic polymeric solution. Acta Biomater..

[39] Bohner M., Baroud G. (2005). Injectability of calcium phosphate pastes. Biomaterials.

[40] Shaw D.H. (2017). Drugs Acting on the Gastrointestinal Tract. Pharmacology and Therapeutics for Dentistry: Seventh Edition.

[41] Oyeneyin B. (2015). Introduction to the Hydrocarbon Composite Production System.

[42] Vu A.A., Burke D.A., Bandyopadhyay A., Bose S. (2021). Effects of surface area and topography on 3D printed tricalcium phosphate scaffolds for bone grafting applications. Addit. Manuf..

[43] El-Bassyouni G.T., Guirguis O.W., Abdel-Fattah W.I. (2013). Morphological and macrostructural studies of dog cranial bone demineralized with different acids. Curr. Appl. Phys..

[44] Murugan R., Ramakrishna S., Panduranga Rao K. (2006). Nanoporous hydroxy-carbonate apatite scaffold made of natural bone. Mater. Lett..

[45] Qashou S.I., El-Zaidia E.F.M., Darwish A.A.A., Hanafy T.A. (2019). Methylsilicon phthalocyanine hydroxide doped PVA films for optoelectronic applications: FTIR spectroscopy, electrical conductivity, linear and nonlinear optical studies. Phys. B Condens. Matter.

[46] Peppas N.A. (1977). Tear propagation resistance of semicrystalline polymeric networks. Polymer (Guildf).

[47] Hassan C.M., Peppas N.A. (2000). Structure and applications of poly(vinyl alcohol) hydrogels produced by conventional crosslinking or by freezing/thawing methods. Adv. Polym. Sci..

[48] Thomas J., Lowman A., Marcolongo M. (2003). Novel associated hydrogels for nucleus pulposus replacement. J. Biomed. Mater. Res. Part A.

[49] Hennink W.E., Van Nostrum C.F. (2002). Novel crosslinking methods to design hydrogels. Adv. Drug Deliv. Rev..

[50] Ma Y., Bai T., Wang F. (2016). The physical and chemical properties of the polyvinylalcohol/polyvinylpyrrolidone/hydroxyapatite composite hydrogel. Mater. Sci. Eng. C.

[51] Harrison J.P., Berry D. (2017). Vibrational spectroscopy for imaging single microbial cells in complex biological samples. Front. Microbiol..

[52] Kobayashi M., Kitaoka Y., Kobayashi M. (1997). Complex formation of boric acids with DI- and TRI- carboxylic acids and poly(vinyl alcohol) in aqueous solutions. Macromol. Symp..

[53] Huang M., Hou Y., Li Y., Wang D., Zhang L. (2017). High performances of dual network PVA hydrogel modified by PVP using borax as the structure-forming accelerator. Des. Monomers Polym..

[54] Barrera J., Rodríguez J., Perilla J., Algecira N. (2007). Estudio de la degradación térmica de poli(alcohol vinílico) mediante termogravimetría y termogravimetría diferencial. Ing. Investig..

[55] Premalatha M., Vijaya N., Selvasekarapandian S., Selvalakshmi S. (2016). Characterization of blend polymer PVA-PVP complexed with ammonium thiocyanate. Ionics (Kiel).

[56] Wang J., Gao C., Zhang Y., Wan Y. (2010). Preparation and in vitro characterization of BC/PVA hydrogel composite for its potential use as artificial cornea biomaterial. Mater. Sci. Eng. C.

[57] Tripathi S.K., Gupta A., Kumari M. (2012). Dielectric and Modulus spectra (Bode)—Studies on electrical conductivity and dielectric behaviour of PVdF–HFP–PMMA–NaI polymer blend electrolyte. Bull. Mater. Sci..

[58] Yang C.C., Lee Y.J., Chiu S.J., Lee K.T., Chien W.C., Lin C.T., Huang C.A. (2008). Preparation of a PVA/HAP composite polymer membrane for a direct ethanol fuel cell (DEFC). J. Appl. Electrochem..

[59] Sun S., Gebauer D., Cölfen H. (2016). A solvothermal method for synthesizing monolayer protected amorphous calcium carbonate clusters. Chem. Commun..

[60] Li L., Yang Y., Lv Y., Yin P., Lei T. (2020). Porous calcite CaCO3 microspheres: Preparation, characterization and release behavior as doxorubicin carrier. Colloids Surf. B Biointerfaces.

[61] Freyman T.M., Yannas I.V., Gibson L.J. (2001). Cellular materials as porous scaffolds for tissue engineering. Prog. Mater. Sci..

[62] Mountziaris P.M., Mikos A.G. (2008). Modulation of the inflammatory response for enhanced bone tissue regeneration. Tissue Eng. Part B Rev..

[63] Minagar S., Lin J., Li Y., Berndt C.C., Wen C. (2017). Nanotopography and surface chemistry of TiO2-ZrO2-ZrTiO4 nanotubular surfaces and the influence on their bioactivity and cell responses. Metallic Foam Bone: Processing, Modification and Characterization and Properties.

[64] De Odontología F. (2014). Universidad Complutense de Madrid Tesis Doctoral un Composite Nuevo de Fosfato Cálcico-Silicato Cálcico Para la Regeneración Ósea: Caracterización Y Comportamiento Memoria Para Optar al Grado de Doctor Presentada Por Lucas Aparicio.

[65] Rai Y., Pathak R., Kumari N., Sah D.K., Pandey S., Kalra N., Soni R., Dwarakanath B.S., Bhatt A.N. (2018). Mitochondrial biogenesis and metabolic hyperactivation limits the application of MTT assay in the estimation of radiation induced growth inhibition. Sci. Rep..

[66] Parmaksiz M., Lalegül-Ülker Ö., Vurat M.T., Elçin A.E., Elçin Y.M. (2021). Magneto-sensitive decellularized bone matrix with or without low frequency-pulsed electromagnetic field exposure for the healing of a critical-size bone defect. Mater. Sci. Eng. C.

[67] Dadgar N., Ghiaseddin A., Irani S., Rabbani S., Tafti S.H.A., Soufizomorrod M., Soleimani M. (2021). Cartilage tissue engineering using injectable functionalized Demineralized Bone Matrix scaffold with glucosamine in PVA carrier, cultured in microbioreactor prior to study in rabbit model. Mater. Sci. Eng. C.

[68] Neves N., Campos B.B., Almeida I.F., Costa P.C., Cabral A.T., Barbosa M.A., Ribeiro C.C. (2016). Strontium-rich injectable hybrid system for bone regeneration. Mater. Sci. Eng. C.

[69] Dorati R., Colonna C., Genta I., De Trizio A., Modena T., Klöss H., Conti B. (2015). In vitro characterization of an injectable in situ forming composite system for bone reconstruction. Polym. Degrad. Stab..

[70] Bencherif S.A., Sands R.W., Bhatta D., Arany P., Verbeke C.S., Edwards D.A., Mooney D.J. (2012). Injectable preformed scaffolds with shape-memory properties. Proc. Natl. Acad. Sci. USA.

[71] Thai V.V., Lee B.T. (2010). Fabrication of calcium phosphate-calcium sulfate injectable bone substitute using hydroxy-propyl-methyl-cellulose and citric acid. J. Mater. Sci. Mater. Med..

[72] Neill R.O., Mccarthy H.O., Montufar E.B., Ginebra M., Wilson D.I., Lennon A., Dunne N. (2017). Acta Biomaterialia Critical review: Injectability of calcium phosphate pastes and cements. Acta Biomater..

[73] Schiller C., Epple M. (2003). Carbonated calcium phosphates are suitable pH-stabilising fillers for biodegradable polyesters. Biomaterials.

[74] Aquino-Martínez R., Artigas N., Gámez B., Rosa J.L., Ventura F. (2017). Extracellular calcium promotes bone formation from bone marrow mesenchymal stem cells by amplifying the effects of BMP-2 on SMAD signalling. PLoS ONE.

[75] Alhashimi R.A., Mannocci F., Sauro S. (2017). Bioactivity, cytocompatibility and thermal properties of experimental Bioglass-reinforced composites as potential root-canal filling materials. J. Mech. Behav. Biomed. Mater..

[76] Al-Wafi R., Eldera S.S., Hamzawy E.M.A. (2020). Characterization and in vitro bioactivity study of a new glass ceramic from mica/apatite glass mixtures. J. Mater. Res. Technol..

[77] Radin S.R., Ducheyne P. (1993). The effect of calcium phosphate ceramic composition and structure on in vitro behavior. II. Precipitation. J. Biomed. Mater. Res..

[78] Yuan H., Barbieri D., Luo X., Van Blitterswijk C.A., De Bruijn J.D. (2017). Calcium phosphates and bone induction. Compr. Biomater. II.

[79] Roberts T.T., Rosenbaum A.J. (2012). Bone grafts, bone substitutes and orthobiologics The bridge between basic science and clinical advancements in fracture healing. Organogenesis.

[80] Laurencin C.T., Jiang T. (2014). Bone Graft Substitutes and Bone Regenerative Engineering.

[81] Adkisson H.D., Strauss-Schoenberger J., Gillis M., Wilkins R., Jackson M., Hruska K.A. (2000). Rapid quantitative bioassay of osteoinduction. J. Orthop. Res..

[82] Glowacki J. (2005). A review of osteoinductive testing methods and sterilization processes for demineralized bone. Cell Tissue Bank..

[83] Barradas A.M.C., Yuan H., van Blitterswijk C.A., Habibovic P. (2011). Osteoinductive biomaterials: Current knowledge of properties, experimental models and biological mechanisms. Eur. Cell. Mater..

[84] Katz J.M., Nataraj C., Jaw R., Deigl E., Bursac P. (2009). Demineralized bone matrix as an osteoinductive biomaterial and in vitro predictors of its biological potential. J. Biomed. Mater. Res. Part B Appl. Biomater..

[85] Pawelec K.M., White A.A., Best S.M. (2019). Properties and characterization of bone repair materials. Bone Repair Biomaterials.

[86] Zhao X., Liang M., Li X., Qiu X., Cui L. (2018). Identification of key genes and pathways associated with osteogenic differentiation of adipose stem cells. J. Cell. Physiol..

[87] Han B., Tang B., Nimni M.E. (2003). Quantitative and sensitive in vitro assay for osteoinductive activity of demineralized bone matrix. J. Orthop. Res..

[88] Zhao M., Dai Y., Li X., Li Y., Zhang Y., Wu H., Wen Z., Dai C. (2018). Evaluation of long-term biocompatibility and osteogenic differentiation of graphene nanosheet doped calcium phosphate-chitosan AZ91D composites. Mater. Sci. Eng. C.

[89] Wang Y., Bian Y., Zhou L., Feng B., Weng X., Liang R. (2020). Biological evaluation of bone substitute. Clin. Chim. Acta.

