# Supplementary figures and images for: Formulation and Characterization of a New Injectable Bone Substitute Composed PVA/Borax/CaCO3 and Demineralized Bone Matrix
Source: J Funct Biomater. 2021 Aug 11;12(3):46. doi: 10.3390/jfb12030046 (PMC8395841; doi:10.3390/jfb12030046)

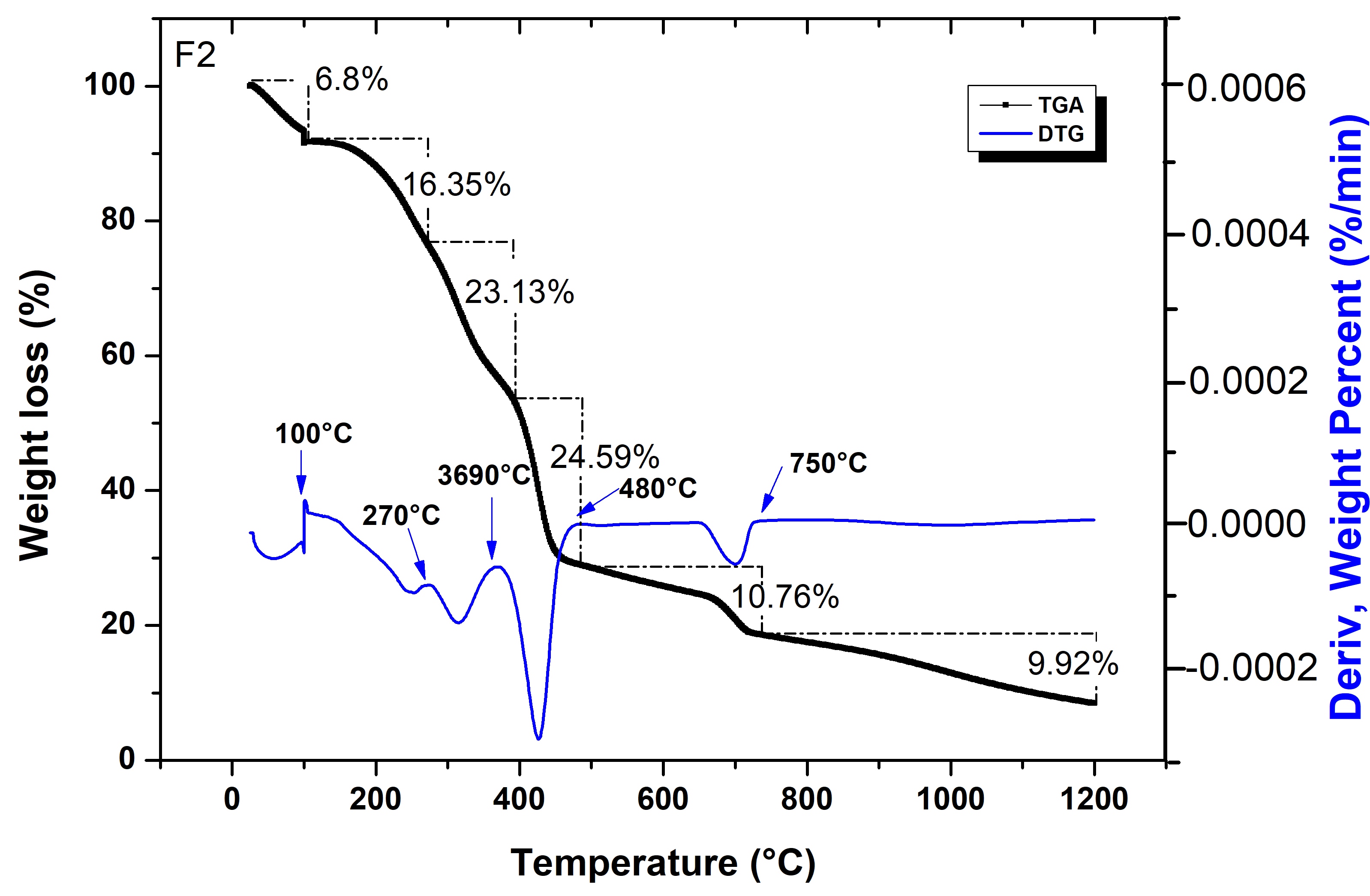

Supplement: Supplementary file 1 [file jfb-12-00046-s001.zip › jfb-1283210-supplementary.jpg]
